# High B-value diffusion tensor imaging for early detection of hippocampal microstructural alteration in a mouse model of multiple sclerosis

**DOI:** 10.1038/s41598-022-15511-0

**Published:** 2022-07-14

**Authors:** Amandine Crombé, Renaud Nicolas, Nathalie Richard, Thomas Tourdias, Bassem Hiba

**Affiliations:** 1 INSERM, U1215, Neurocentre Magendie, 33000 Bordeaux, France; 2 University of Bordeaux, 33000 Bordeaux, France; 3 Aquitaine Institute for Cognitive and Integrative Neuroscience (INCIA), UMR 5287, CNRS, Université de Bordeaux, Bordeaux, France; 4 Département de R&D, Institut Ostéopathique Animalier, 33170 Gradignan, France; 5 Institute of Cognitive Neuroscience Marc Jeannerod, CNRS/UMR 5229, 69500 Bron, France; 6 Université Claude Bernard, Lyon 1, 69100 Villeurbanne, France; 7 Department of Neuroradiology, Pellegrin University Hospital, 33000 Bordeaux, France

**Keywords:** Biophysics, Neuroscience, Anatomy, Biomarkers, Medical research, Neurology

## Abstract

Several studies have highlighted the value of diffusion tensor imaging (DTI) with strong diffusion weighting to reveal white matter microstructural lesions, but data in gray matter (GM) remains scarce. Herein, the effects of b-values combined with different numbers of diffusion-encoding directions (NDIRs) on DTI metrics to capture the normal hippocampal microstructure and its early alterations were investigated in a mouse model of multiple sclerosis (experimental autoimmune encephalomyelitis [EAE]). Two initial DTI datasets (B2700-43Dir acquired with b = 2700 s.mm^−2^ and NDIR = 43; B1000-22Dir acquired with b = 1000 s.mm^−2^ and NDIR = 22) were collected from 18 normal and 18 EAE mice at 4.7 T. Three additional datasets (B2700-22Dir, B2700-12Dir and B1000-12Dir) were extracted from the initial datasets. In healthy mice, we found a significant influence of b-values and NDIR on all DTI metrics. Confronting unsupervised hippocampal layers classification to the true anatomical classification highlighted the remarkable discrimination of the molecular layer with B2700-43Dir compared with the other datasets. Only DTI from the B2700 datasets captured the dendritic loss occurring in the molecular layer of EAE mice. Our findings stress the needs for both high b-values and sufficient NDIR to achieve a GM DTI with more biologically meaningful correlations, though DTI-metrics should be interpreted with caution in these settings.

## Introduction

Diffusion Magnetic Resonance Imaging (dMRI) refers to Magnetic Resonance Imaging (MRI) acquisition sensitized to the diffusion of water molecules in the tissues of interest^1–3^. As water molecule random motion is influenced by the presence of cellular microstructures, dMRI is a unique technique allowing non-invasive investigation of the micro-organization of living tissues^3^.

The characteristics of the microstructure of living tissues, deduced from dMRI data, depends on the parameterization of the diffusion encoding of the water molecules (diffusion time, diffusion encoding magnetic field gradients intensities and directions) and also on the modeling of the dMRI signal^1,3,4^. Diffusion tensor imaging (DTI) models the diffusivity of water molecules in each voxel as a Gaussian ellipsoid and provides quantitative maps of water diffusivity such as fractional anisotropy (FA), axial diffusivity (AD, water diffusivity along tracts), radial diffusivity (RD, water diffusivity perpendicular to tracts) and mean diffusivity (MD)^2,5^. DTI has been widely recognized for its ability to characterize in each voxel the oriented diffusivity of water molecules occurring within the anisotropic micro-organization of cerebral white matter (WM), for its quick and easy computing, for its robustness and for its generalized scientific and medical usage^6,7^.

dMRI is typically acquired with a diffusion weighting factor b-value of 1000 s.mm^−2^, but could also be achieved with higher sensitivities to the water diffusion process using higher b-values. Several reports have highlighted a substantial gain in dMRI ability to investigate a wide panel of brain diseases inducing focal lesions when high b-value is used. For example, high b-value dMRI appears useful for detecting stroke^8–10^, Wallerian degenerations and encephalopathies^7,11^, and for discriminating high-grade and low-grade gliomas^9^.

Several reports have explored the value of dMRI with high diffusion-weighting to identify non-focal (diffuse) lesions of the brain microstructure. For example, Baumann et al. have shown in human that the sensitivity of DTI to detect the effects of schizophrenia in pre-frontal lobes white matter was increased with b = 4000 s.mm^−2^ rather than b = 1000 s.mm^−2^^12^. In animals, dMRI with high b-values could also improve the detectability of the WM demyelination process^13^. The myelination process occurring during brain maturation have also been reported to be better detected with DTI obtained with b = 2500 s.mm^−2^ rather than with b = 1000 s.mm^−2^^14^. Nevertheless, these studies focused on the physiological or pathophysiological conditions that induce relatively important changes in brain microstructure, such as myelination or demyelination, and only examined brain WM without or with limited consideration of the grey matter (GM). Therefore, little is known about the precise added value of strong b-values versus standard b-values to characterize GM microarchitecture and to early detect pathological alterations. Furthermore, the impact of the number of diffusion directions (NDIR), combined with high b-values to encode the water molecules diffusivity in GM, has been poorly investigated.

Yet, preliminary researches have recently demonstrated that DWI with high b-value could detect precocious and subtle pathological processes occurring in GM. Indeed, using the Experimental Autoimmune Encephalomyelitis (EAE) mouse model of multiple sclerosis^15^, DTI with b = 2700 s.mm^−2^ and 43 diffusion-encoding directions was able to detect significant decreases in axial diffusivity and mean diffusivity in the molecular layer of the dentate gyrus, which correlated with an early decrease in dendritic arborization responsible for cognitive impairment in the EAE mice^16,17^.

Importantly, strong diffusion-weighting reduces the Signal-to-Noise Ratio (SNR) of the dMRI and exacerbates its sensitivity to various artifacts. Therefore, the achievement of dMRI with a high diffusion weighting, with a sufficient spatial-resolution to distinguish the mouse hippocampal layers and with a high enough SNR to accurately reconstruct the diffusion tensor maps becomes a very challenging issue. To obtain these results in mouse hippocampus, a 3D multi-shot Echo Planar imaging (3D-msEPI) pulse sequence was used in this study as an alternative to the standard 2D signal-shot EPI pulse sequence to collect dMRI data^18^.

Thus, the aim of this study was to challenge our initial results obtained in vivo in the hippocampus of EAE and control mice to know whether high b-value and high NDIR were really mandatory to capture histologically proven alterations. To do so, we down-sampled the diffusion encoding schema to reconstruct different DTI datasets (b = 2700 s.mm^−2^ with NDIR = 22; b = 2700 s.mm^−2^ with NDIR = 12; and b = 1000 s.mm^−2^ with NDIR = 12) in addition to the initial ones (b = 2700 s.mm^−2^ with NDIR = 43; and b = 1000 s.mm^−2^ with NDIR = 22), and we subsequently investigated their abilities to discriminate the different hippocampal layers, as well as to detect early microarchitectural alterations in the molecular layer of EAE mice.

## Materials and methods

### Animal model

The raw experimental data used in this study have been differently processed in another study from our group, which compared DTI and Neurite Orientation Dispersion and Density Imaging (NODDI) models^17^. Briefly, 7-to-9 week old female C57BL6/J mice (Janvier Labs, Le Genest Saint Isle, France) were injected subcutaneously at the base of the tail with 200 μg of Myelin Oligodendrocyte Glycoprotein peptide 35–55 (MOG35-55, AnaSpec, Fremont, CA, USA) emulsified in 200 μL of Complete Freund’s adjuvant (CFA, BD Difco, Franklin Lakes, NJ, USA) containing 6 mg/mL of desiccated Mycobacterium tuberculosis (H37Ra, BD Difco). Animals received intraperitoneal injections of Pertussis Toxin (Sigma-Aldrich, St. Louis, MO, USA) on the day of immunization and 2 days later (300 ng/injection). Control mice were injected with 200 μL of CFA emulsified phosphate-buffer saline. All animals were weighted daily and scored for clinical symptoms using the standard grading scale: 0: unaffected, 1: flaccid tail, 2: hind limb weakness and/or ataxia, 3: hind limb paralysis, 4: paralysis of all four limbs and 5: moribund. A total of 18 control mice and 18 EAE mice were imaged in vivo with dMRI 20 days after immunization and prior to sacrifice for histological quantification of neurites and glial markers in each hippocampal layer^17^. All animal care and experiments were conducted in accordance with the European Directive (2010/63/EU) and the ‘Animal Research: Reporting of In Vivo Experiments’ (ARRIVE) guidelines, and after approval of the Bordeaux University ethical committee (approval number 02046.01)^19^.

### dMRI data

MRI acquisitions were performed on a 4.7 Tesla MR-system (Biospec 47/20, Bruker BioSpin MRI, Ettlingen, Germany), equipped with a high-performance magnetic gradient field system (capable of 680 mT/maximum strength and 110 μs rise time). A volume coil for radiofrequency pulse transmission and a mouse-head four-element phased array coil for magnetic-resonance (MR)-signal detection were used. Mice were anesthetized with isoflurane in air and, in order to reduce motion artifacts, were carefully placed in a head holder with ear bars. Body temperature was kept at 37 °C with a water circulation heating bed. Respiration was monitored throughout the scan.

A 3D-ms-EPI was used in order to perform a 3D sampling of the Fourier-space to increase SNR and resolution. Two sets of dMRI data were collected per mouse. For the first data set (B1000-images), 22 diffusion-weighted (DW) coronal images were acquired with b = 1000 s.mm^−2^ applied along 22 non-collinear diffusion encoding directions (intensity of the diffusion gradients [G] = 340 mT/m, duration of each diffusion gradient pulse [δ]/time interval separating diffusion pulses [Δ] = 3.2/14 ms), while 43 DW coronal images with b = 2700 s.mm^−2^ applied along 43 non-collinear diffusion encoding directions were acquired for the second data-set (B2700-images, G = 559 mT/m, δ/Δ = 3.2/14 ms)^20^. Two non-DW imaging (i.e., b = 0 s.mm^−2^, named B0-images) were also collected for each dataset.

The other acquisition parameters were: Echo-Time (TE)/Repetition-Time (TR) = 38/2000 ms, matrix = 196 × 148 × 32 pixels, spatial resolution = 82 × 81 × 203 μm^3^ and Field-of-View = 16 × 12 × 6.5 mm^3^ including the whole hippocampus in the rostro-caudal axis. The 3D-msEPI readout module was used with two segments in brain superior-inferior orientation (K_y_) and with a phase encoding in brain posterior-anterior orientation (K_z_). The K_y_ lines were acquired through 2 interleaves, and each interleaf was sampled during one of the EPI shots to reduce echo-train length and echo-time. For a given K_z_ encoding step, all the in-plane (K_x_, K_y_) interleaves were acquired successively before proceeding to next K_Z+1_ encoding step. A partial scan of k-space was applied in K_y_ to further decrease echo-train length and in K_z_ to reduce acquisition time. A high order shim procedure was performed before the scan to optimize the static magnetic field homogeneity. The total acquisition time was 1h50 min per mouse.

### dMRI pre-processing

FSL diffusion tool software (https://fsl.fmrib.ox.ac.uk/fsl/fslwiki/FDT) was used to pre-process the dMRI data. First, eddy currents and K_y_-distortions effects were corrected using the eddy function^21^. All B0-images, the 22 DW-images acquired with b = 1000 s.mm^−2^ (B1000-images) and the 43 DW-images acquired with b = 2700 s.mm^−2^ (B2700-images) were co-registered with one of the B0-images before being divided again into two datasets.

In order to obtain DTI datasets with similar NDIR at b = 2700 s.mm^−2^ and at b = 1000 s.mm^−2^, a home-made procedure was applied to discard 21 of the 43 B2700-images and to derive a new DTI dataset with 22 B2700-images. This procedure relied in the following four steps:

(i) computing the angle values ‘*d*’ separating each diffusion-vector from all the other vectors of the data-set;
(ii) counting the number of vectors located in the close neighborhood (*d* < *30°*) of each diffusion-vector;
(iii) removing the diffusion-vector exhibiting the largest number of close neighbors and its corresponding DW-image from the DTI data-set;
(iv) recursively applying steps (i), (ii) and (iii) to the remaining diffusion-vectors until reaching the desired diffusion-direction number.

The same procedure was used to derive a DTI dataset with 12 B2700-images and a DTI dataset with 12 B1000-images. Supplementary Data S1 lists the b-values and the diffusion directions vectors used in this study.

To summarize, the following datasets were reconstructed and analyzed in this study: (1) b = 2700 s.mm^−2^ with NDIR = 43 (B2700-43Dir); (2) b = 2700 s.mm^−2^ with NDIR = 22 (B2700-22Dir); (3) b = 2700 s.mm^−2^ with NDIR = 12 (B2700-12Dir); (4) b = 1000 s.mm^−2^ with NDIR = 22 (B1000-22Dir); and (5) b = 1000 s.mm^−2^ with NDIR = 12 (B1000-12Dir).

For each of these five datasets, diffusion tensor maps (the principal eigenvector [V1, V2 and V3], the eigenvalues [λ1, λ2 and λ3], FA, MD, AD and RD) and the fit residue as the sum-of-square error (SSE) were computed by using *dtifit* FSL function.

### dMRI data quality control

A rigorous quality control was carried out by two blinded experts at the different stages of the data processing. First, the original images and the eddy-current corrected images were checked slice-by-slice to depict any potential effects of eddy currents, EPI ghosting and signal losses. The presence of artifacts in the hippocampal region was visually and carefully inspected. Second, another quality control was performed on diffusion tensor maps. The residual of the DTI fit was also checked for each dataset.

### Data analysis

**Figure 1:**
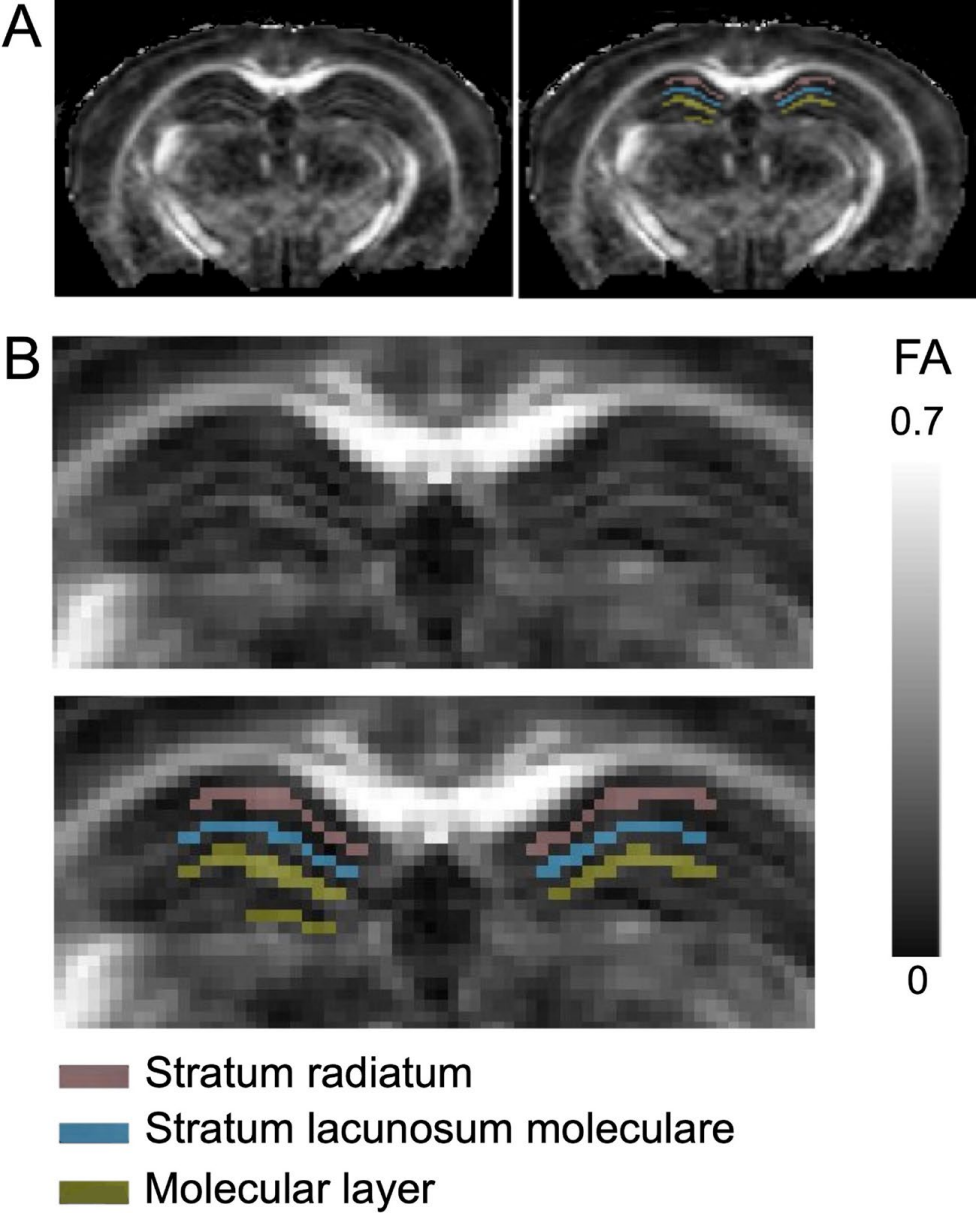
Example of brain fractional anisotropy (FA) parametric maps (**A**) with a magnification (**B**) on the hippocampus with and without the segmentations of the three volumes of interest corresponding to the three main hippocampal layers (i.e., Stratum Radiatum, Stratum Lacunosum Molecular and Molecular Layer).

The volumes of interest (VOIs) were manually delineated on three consecutive slices of FA maps superimposed on color-coded V1 maps from the B2700-43Dir dataset covering the dorsal part of the hippocampus. Three VOIs were delineated for the three main hippocampal layers: (i) the *stratum radiatum* (SR) of *cornu ammonis* subfield 1 (CA1), (ii) the *stratum lacunosum moleculare* (SLM) and (iii) the molecular layer (ML) of the dentate gyrus. The VOIs were drawn by a neuroradiologist blinded from the groups of animals and validated by a second neuroradiologist. Figure 1 shows an example of the anatomical dispositions and segmentations on the FA parametric map. Additionally, a VOI was drawn on the corpus callosum (CC) on the same three consecutive slices in order to estimate the SSE in the WM. After propagating the VOIs on the other parametric maps of all coregistered datasets and checking the correct locations, the mean values of each of DTI metrics were extracted for each layer and each animal using the *fslstats* FSL function.

In order to assess the SNR of each of the 5 dMRI datasets, a background VOI was manually delineated for each mouse in an artifact-free air region as close as possible to the hippocampus. The SR, CA1, ML and background VOIs were propagated on all co-registered images of each dMRI dataset.

The B0-image SNR was computed for each DW-dataset as the ratio between the average signal intensity (SI) measured in the SR, CA1 and ML VOIs and the standard deviation (SD) of the noise measured in the B0-image background^22^. The global SNR was then computed for each dMRI dataset as the average of the SNRs of all its DW-images, as follows:

$${SNR}_{global}=\frac{{\sum }_{i=1}^{NDIR}\frac{averageSI\left(hippocampus,i\right)}{SD\left(background,i\right)}}{NDIR}$$

Finally, the contrast-to-noise ratio (CNR) maps were automatically computed for each dataset using the FSL 6.0 « QUAD (QUality Assessment for DMRI) » tool (https://git.fmrib.ox.ac.uk/matteob/eddy_qc_release.git)^21,23,24^. For each dMRI dataset, the CNR map provides a global quantification of the amount of angular contrast generated between non-B0 images by applying diffusion vectors with different spatial directions. Note that the used script has failed to compute the CNR map for the B1000-12Dir dataset. SR, CA1 and ML VOIs were then used to compute the mean CNR values for the hippocampus and for each of the SR, CA1 and ML layers.

### Diffusion kurtosis imaging (DKI)

State-of-the-art DKI was performed with the free software Diffusional Kurtosis Estimator (https://www.nitrc.org/dke/ from Medical University of South Carolina, Center for Biomedical Imaging)^25^. Weighted linear least-squares constrained with minimal K_mean_ = 0 and maximal K_mean_ = 3, as recommended in the study by Tabesh et al., was used^26^. Image smoothing was not applied. Calculation of DKI-related FA, axial diffusivity (D_ax_), radial diffusivity (D_rad_), mean diffusivity (D_mean_), axial excess kurtosis (K_ax_), radial excess kurtosis (K_rad_), mean excess kurtosis (K_mean_), mean of the kurtosis tensor (MKT) and Kurtosis Fractionnal Anisotropy (KFA) were computed with DKE. Average DKI quantitative values were extracted from the VOIs using the fslstats program, a part of FSL^27^. Supplementary Data S2 shows the DKI-related generated images.

### Statistical analysis

The statistical analyses were performed with R (v4.1.0, R Foundation for Statistical Computing, Vienna, Austria). All tests were two-tailed. A p-value less than 0.05 was deemed significant. P-values were adjusted for multiple comparisons in post-hoc comparisons using the Benjamini, Hochberg, and Yekutieli procedure (which controls the false discovery rate and the expected proportion of false discoveries amongst the rejected hypotheses)^28^.

#### Quality control

The angles separating the diffusion encoding vectors from their closest neighbor (named minimum angles) were extracted for each DTI dataset and compared by using one-way analysis of variance (ANOVA) with post-hoc unpaired t-test (or non-parametric Kruskal–Wallis test with post-hoc unpaired Wilcoxon test, as appropriate). Post-hoc tests were used to check the comparability of the 12 vectors from B1000-12Dir and B2700-12Dir, and of the 22 vectors from B1000-22Dir and B2700-22Dir.

The SNR, the CNRs, the SSE in the hippocampus and the SSE in the CC were compared across the five datasets by using one-way repeated-measures ANOVA (with Greenhouse–Geisser correction if needed) and post-hoc pairwise t-tests, or non-parametric Friedman test and post-hoc pairwise Wilcoxon tests, as appropriate. Furthermore, the difference between the SSE in the hippocampus and in the CC was compared for each dataset with adjusted pairwise Wilcoxon tests.

#### Discriminations of the hippocampal layers in healthy mice

First, for each of the five datasets, we compared the mean values of AD, FA, MD and RD extracted from the three hippocampal layers of the control mice by using repeated-measures one-way ANOVA (with post-hoc pairwise t-test) or non-parametric repeated-measures Friedman test (with post-hoc pairwise Wilcoxon test), as appropriate. The effect size, which represents the total variability that is accounted for by the variation of the dependent variable, was estimated for each ANOVA and Friedman test, by using general eta squared (η2, categorized as large when > 0.140) and Kendall’s W coefficient (categorized as large when > 0.800), respectively^29^. Second, we evaluated and compared the ability of the combinations of b-values and NDIR from the different datasets to discriminate the three hippocampal layers. To do so, we developed five unsupervised classifications of the layers relying on the DTI metrics coming from the five datasets by using the k-medoid clustering technique and the partition around medoid algorithm (parameters were set to k = 3 with the Euclidean distance)^30^. Then, we compared these five classifications with the true hippocampal anatomy by using the adjusted Rand index (ARI), which measures the similarity between two classifications of the same objects (herein the hippocampal layers) by the proportions of agreements between the two partitions from the “ClusterR” R package^31^. The ARI ranges from − 1 (independent labeling) to + 1 (perfect labeling).

#### Detections of the GM microstructural alterations induced by EAE in the molecular layer

Comparison between the EAE and the control mice was performed by using unpaired homo- or heteroscedastic t-tests or unpaired Wilcoxon tests, as appropriate. This comparison was investigated separately for each dataset and each diffusion metric. No P-value adjustment was performed in this part, in agreement with the methods proposed in the study by Crombé et al. since the authors validated their findings with biological correlations^17^. Since it can be argued that DTI is not the reference model in high b-value settings and that DKI may be more appropriate, we also compared the discriminative performances of DTI metrics from the different datasets to those of DKI metrics.

## Results

### Quality control—SNR and CNR

Table 1 summarizes the mean values of the minimal angles separating the diffusion-encoding vectors, the SNR and CNRs for the five DTI datasets, with statistical comparisons (more post-hoc tests are represented in Supplementary Data S3).

**Table 1:** Assessment of the influence of the diffusion tensor imaging (DTI) dataset on measurements of image quality, i.e. signal-to-noise (SNR) ratio, contrast-to-noise ratios (CNR) and minimal angles separating the diffusion encoding vectors.

Measurements	Datasets						F-value	Size effect	P-value	Post-hoc tests adjusted P-value	
	B0	B1000-12Dir	B1000-22Dir	B2700-12Dir	B2700-22Dir	B2700-43Dir				B1000-12Dir vs. B2700-12Dir	B1000-22Dir vs. B2700-22Dir
Minimum angle (°)	–	38.4 ± 10.2	29.3 ± 10.1	38.1 ± 5.4	24.5 ± 7.5	20 ± 4.7	58.03^§^	0.510	< 0.0001***	1	0.0728
SNR in hippocampus	79.5 ± 7.7	47.2 ± 3.7	47.2 ± 3.6	25 ± 1.8	24.7 ± 1.9	25.1 ± 1.9	2008.17	0.961	< 0.0001***	< 0.0001***	< 0.0001***
CNR in hippocampus	–	–	1.4 ± 0.5	1.7 ± 1.2	1.7 ± 0.8	1.6 ± 0.5	2.47	0.021	0.1040	–	–
CNR in ML	–	–	1.5 ± 0.5	1.8 ± 1.3	1.9 ± 1	1.8 ± 0.6	3.78	0.031	0.0340*	–	0.0110*
CNR in SLM	–	–	1.3 ± 0.4	1.7 ± 1.2	1.6 ± 0.8	1.4 ± 0.5	4.59	0.049	0.2047	–	–
CNR in SR	–	–	1.3 ± 0.5	1.5 ± 1	1.4 ± 0.6	1.4 ± 0.5	0.824	0.007	0.4230	–	–

Measurements in each dataset are expressed as mean ± standard deviation. F-values and P-values correspond to one-way repeated measures analysis of variance, except for the minimal angle (§) which corresponds to Kruskal–Wallis test (non-parametric, non repeated test). The CNRs for the B1000-12Dir was incalculable according to the FSL “Eddy” script.

*P < 0.05; **P < 0.005; ***P < 0.001.

*B* b-value, *Dir* number of diffusion gradient directions, *ML* molecular layer, *SLM* stratum lacunosum moleculare, *SR* stratum radiatum.

No significant difference was found between the minimal angles separating the diffusion encoding vectors from the B1000-22Dir dataset and those of the B2700-22Dir dataset (P = 0.0728). Similarly, the minimal angles separating the diffusion encoding vectors of the B1000-12Dir and the B2700-12Dir datasets were not significantly different (P = 1).

The quality control revealed EPI ghosts and/or MR-signal dropout in two EAE and three control mice. These artifacts were probably due to cardiovascular-pulsations or respiratory movements produced by an insufficient immobilization of the mouse head. Therefore, these artifacted data were excluded and only data from 16 EAE and 15 control mice were analyzed in the remaining of the study. The high quality/resolution of all the other approved DW-images allowed to identify and manually delineate the three main hippocampal layers (Fig. 1).

**Figure 2:**
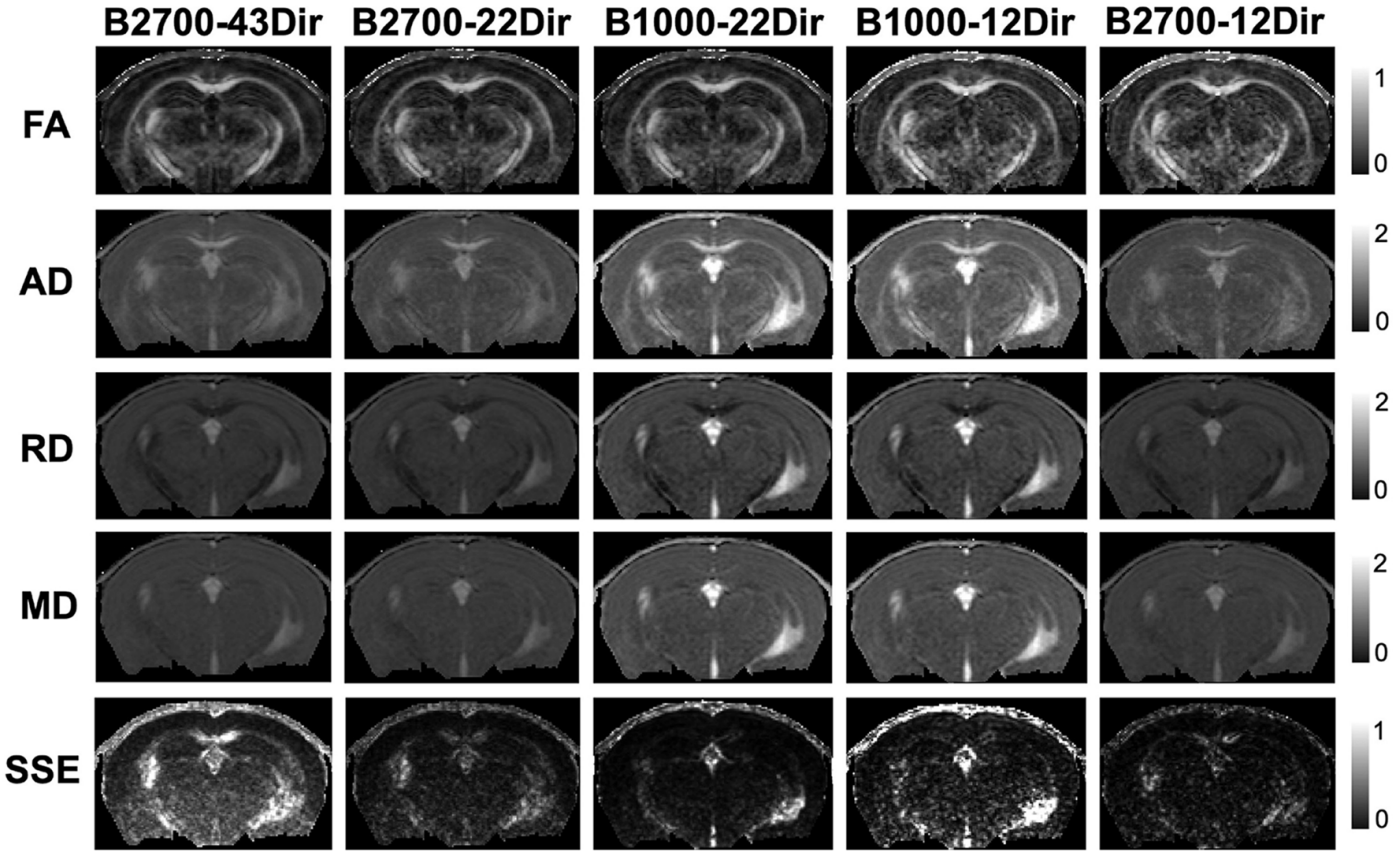
Coronal views of the four brain parametric maps and sum of square residual (SSR) after applying the DTI model, reconstructed using each of the five-diffusion tensor imaging (DTI) datasets in a same control mouse at a spatial-resolution of 82 × 81 × 200 µm^2^. All diffusivity maps (axial, mean and radial diffusivities) are presented in µm^2^/ms. *AD* axial diffusivity, *B* b-value, *Dir* number of diffusion encoding directions, *FA* fractional anisotropy, *MD* mean diffusivity, *RD* Radial Diffusivity, *SSE* sum of square error.

Qualitatively, decreasing the number of diffusion-encoding directions from 43 to 22 and then to 12 did not affect the SNRs of DW-datasets but reduced the overall quality of diffusion-tensor maps, which appeared noisier (Fig. 2). Furthermore, b-value increasing from 1000 to 2700 s.mm^−2^ visually led to darker DTI maps (Fig. 2). The map of the SSE demonstrated higher values when the b-value increased suggesting a decrease in the DTI model fitting accuracy.

The average SNR assessed in the hippocampus ranged from 24.7 ± 1.9 for B2700-22Dir to 47.2 ± 3.6 for B1000-22Dir and 47.2 ± 3.7 for B1000-12Dir. As previously reported, we found that the SNR was significantly influenced by the diffusion weighting (b-value) of the DTI dataset with a large size effect (P < 0.0001, η^2^ = 0.961). Post-hoc tests showed that the SNR in the hippocampus was significantly lower with B2700-12Dir compared with B1000-12Dir (P < 0.0001), as well as with B2700-22Dir compared with B1000-22Dir (P < 0.0001).

There was a significant influence of the DTI dataset on the CNR in the ML, but not in the SLM and SR—with all size effects remaining weak (P = 0.0340 [η^2^ = 0.031], 0.2047 [W = 0.049] and 0.4230 [η^2^ = 0.007], respectively). Indeed, the CNR in the ML was the highest in B2700-22Dir dataset (1.9 ± 1 versus 1.5 ± 0.5 in B1000-22Dir, P = 0.0110).

For both CC and hippocampus, the DTI dataset had a significant influence on the SSE with a large size effect (F = 121.8, W = 0.983, P < 0.0001, and F = 110.6, η^2^ = 0.892, P < 0.0001, respectively, Fig. 2). In the hippocampus, the post-hoc tests indicated that a significant increase in SSE was observed when the b-value increased for the same NDIR, as well as when the NDIR increased for a fixed b-value (P < 0.0001 for all post-hoc tests). However, in the CC, at b = 1000 s.mm^−2^, the SSE significantly decreased from 12 to 22 directions (P < 0.0001), whereas the SSE gradually and significantly increased while increasing the NDIRs at b = 2700 s.mm^−2^ (P < 0.0001 for all post-hoc tests). Furthermore, the SSE was systematically significantly higher in the CC compared with the hippocampus (all P-values < 0.0001) and the highest difference was found in the B2700-43Dir dataset (Supplementary Data S4).

### Influence of b-value and NDIR on the classification of the hippocampal layers in healthy mice

The visual observations translated quantitatively with decreases in the DTI metrics in the three layers of the hippocampus when increasing the b-values (Table 2). For each layer, the values of the DTI metrics were significantly influenced by the DTI dataset with large size effects (all P-values < 0.0001, range of size effect: η^2^ = 0.497 [for FA in the ML] to W = 1 [for AD in the SR], Supplementary Data S5).

**Table 2:** Assessment in healthy mice of the differences in diffusion tensor imaging (DTI) metrics in the three main hippocampal layers depending on the DTI dataset.

DTI metrics	Dataset	F-value	Size effect	P-value	Metrics in SR	Metrics in SLM	Metrics in ML	Post-hoc tests adjusted P-values		
								SR versus SLM	SR versus ML	SLM versus ML
**AD**	B1000-12Dir	4.5	0.045	0.0210*	0.699 ± 0.036	0.715 ± 0.034	0.716 ± 0.041	0.064	0.043*	1
	B1000-22Dir	7.5	0.067	0.0030**	0.662 ± 0.036	0.683 ± 0.028	0.676 ± 0.038	0.011*	0.016*	0.611
	B2700-12Dir	9.6	0.095	0.0007***	0.563 ± 0.033	0.546 ± 0.027	0.568 ± 0.027	0.012*	0.764	0.004**
	B2700-22Dir	14.4^§^	0.480	0.0007***	0.521 ± 0.024	0.519 ± 0.022	0.535 ± 0.022	1	0.002**	0.002**
	B2700-43Dir	29.9	0.154	< 0.0001***	0.53 ± 0.025	0.519 ± 0.022	0.542 ± 0.024	0.001**	0.002**	< 0.0001***
**RD**	B1000-12Dir	20.8^§^	0.693	< 0.0001***	0.528 ± 0.026	0.535 ± 0.032	0.475 ± 0.026	0.173	0.0003***	0.0003***
	B1000-22Dir	22.5^§^	0.751	< 0.0001***	0.517 ± 0.023	0.518 ± 0.029	0.466 ± 0.023	1	0.0002***	0.0002***
	B2700-12Dir	152.7	0.614	< 0.0001***	0.438 ± 0.018	0.426 ± 0.019	0.391 ± 0.009	< 0.0001***	< 0.0001***	< 0.0001***
	B2700-22Dir	143.2	0.555	< 0.0001***	0.43 ± 0.022	0.417 ± 0.022	0.381 ± 0.012	< 0.0001***	< 0.0001***	< 0.0001***
	B2700-43Dir	171.4	0.546	< 0.0001***	0.432 ± 0.022	0.423 ± 0.022	0.383 ± 0.015	0.0003***	< 0.0001***	< 0.0001***
**MD**	B1000-12Dir	20.8^§^	0.693	< 0.0001***	0.585 ± 0.028	0.595 ± 0.031	0.555 ± 0.027	0.015*	0.002**	0.000335
	B1000-22Dir	22.7_§_	0.756	< 0.0001***	0.565 ± 0.026	0.573 ± 0.028	0.536 ± 0.026	0.044*	0.004**	0.004**
	B2700-12Dir	51.5	0.254	< 0.0001***	0.474 ± 0.022	0.464 ± 0.023	0.446 ± 0.014	0.0002***	< 0.0001***	0.0002***
	B2700-22Dir	76.6	0.278	< 0.0001***	0.46 ± 0.022	0.451 ± 0.021	0.432 ± 0.014	< 0.0001***	< 0.0001***	< 0.0001***
	B2700-43Dir	83.1	0.264	< 0.0001***	0.465 ± 0.023	0.455 ± 0.022	0.436 ± 0.016	< 0.0001***	< 0.0001***	< 0.0001***
**FA**	B1000-12Dir	61.3	0.695	< 0.0001***	0.194 ± 0.019	0.203 ± 0.022	0.267 ± 0.025	0.381	< 0.0001***	< 0.0001***
	B1000-22Dir	54.7	0.702	< 0.0001***	0.173 ± 0.016	0.193 ± 0.021	0.24 ± 0.019	0.01*	< 0.0001***	0.0002
	B2700-12Dir	55.3	0.669	< 0.0001***	0.187 ± 0.024	0.181 ± 0.016	0.249 ± 0.026	0.711	< 0.0001***	< 0.0001***
	B2700-22Dir	28.1^§^	0.938	< 0.0001***	0.133 ± 0.011	0.157 ± 0.019	0.213 ± 0.016	0.0006***	0.0002***	0.0002***
	B2700-43Dir	164.1	0.837	< 0.0001***	0.139 ± 0.011	0.144 ± 0.019	0.216 ± 0.017	0.537	< 0.0001***	< 0.0001***

Metrics are expressed as mean ± standard deviation. F-values and P-values correspond to one-way repeated measures analysis of variance, or repeated measures Friedman test (§).

*P < 0.05; **P < 0.005; ***P < 0.001. Diffusivities are expressed in μm^2^.ms^−1^.

*AD* axial diffusivity, *B* b-value, *Dir* number of diffusion gradient directions, *FA* fractional anisotropy, *MD* mean diffusivity, *ML* molecular layer, *RD* radial diffusivity, *SLM* stratum lacunosum moleculare, *SR* stratum radiatum.

Regarding FA, this metrics decreased when increasing the b-value and the differences were all significant for 22 directions (P-value range: 0.0003–0.0004). Moreover increasing NDIR for fixed b-values also decreased the FA whatever the layers, with significant findings (P-value range: 0.0003–0.012)—except for the comparisons between 22 and 43 directions at b = 2700 s.mm^−2^ in the ML and SR where the FA was similar or increased (Supplementary Data S6).

**Figure 3:**
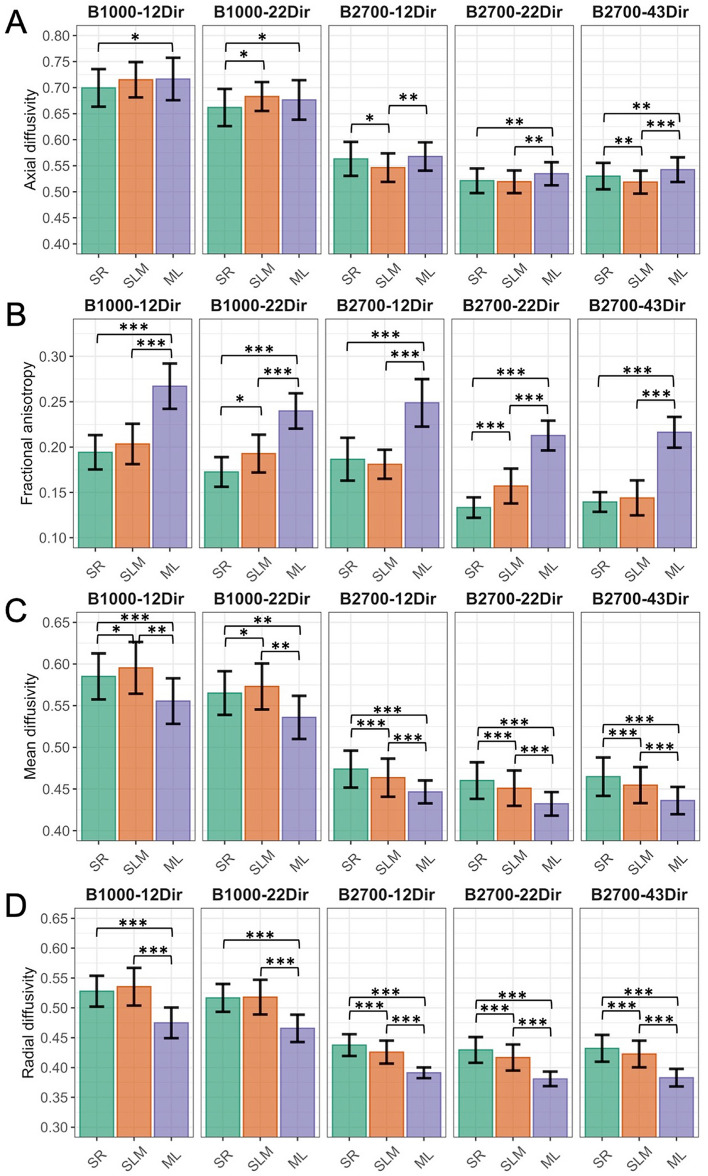
Comparisons of diffusion tensor imaging (DTI) metrics in the three main hippocampal layers of healthy mice depending on the DTI dataset: (**A**) axial diffusivity (AD), (**B**) fractional anisotropy (FA), (**C**) mean diffusivity (MD) and (**D**) radial diffusivity (RD). AD, MD and RD are expressed in μm^2^.ms^−1^. *B* b-value, *Dir* number of diffusion gradient direction, *ML* molecular layer, *SLM* stratum lacunosum moleculare, *SR* stratum radiatum. *P < 0.05; **P < 0.005; ***P < 0.001.

Table 2 and Fig. 3 show the comparisons of each DTI metrics over the SR, SLM and ML depending on the DTI dataset. In all cases, the characteristics of the dataset significantly influenced the DTI metrics in the layers (range of P-values: < 0.0001–0.0210). However, the size effects were moderate instead of large for the following combinations: AD in the B1000-12Dir dataset (η^2^ = 0.045), AD in the B1000-22Dir dataset (η^2^ = 0.067), AD in the B2700-12Dir dataset (η^2^ = 0.095), AD in the B2700-22Dir dataset (W = 0.480), RD in the B1000-12Dir dataset (W = 0.693), RD in B1000-22Dir dataset (W = 0.751), MD in the B1000-12Dir dataset (W = 0.693) and MD in the B1000-22Dir dataset (W = 0.756). In other words, the influence of the DTI dataset on the metrics values across the hippocampal layers was less pronounced for lower b-values and lower NDIRs.

The best discriminations of the hippocampal layers (i.e. all post-hoc tests showing statistical difference with adjusted P-values < 0.0001) were found with the MD and the B2700-22Dir (size effect η^2^ = 0.278, i.e. large) and B2700-43Dir datasets (size effect η^2^ = 0.264, i.e. large). On the contrary, the worst discrimination was obtained with the AD with the B1000-12Dir (lowest size effect η^2^ = 0.045, i.e. moderate effect).

**Figure 4:**
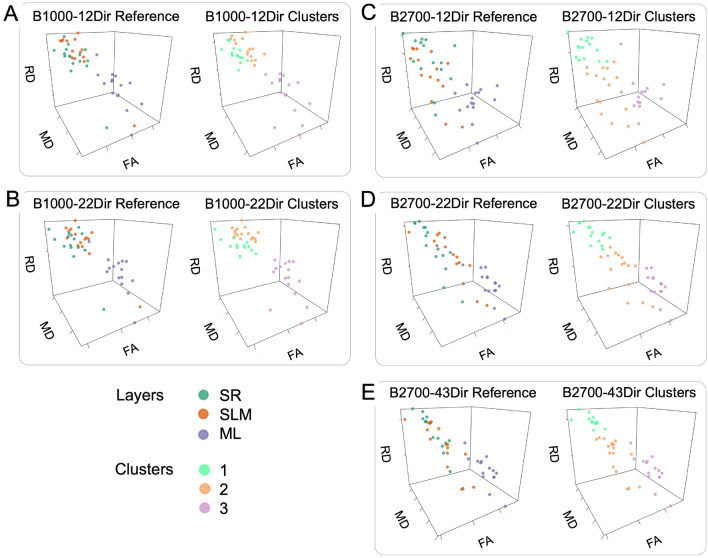
Comparative views of the unsupervised clustering results and the real anatomical classification for each diffusion tensor imaging (DTI) dataset: (**A**) B1000-12Dir, (**B**) B1000-22Dir, (**C**) B2700-12Dir, (**D**) B2700-22Dir, and (**E**) B2700-43Dir. Each point corresponds to one segmentation, characterized by its DTI metrics and is plotted with the same three axes: x: fractional anisotropy (FA), y: mean diffusivity (MD), z: radial diffusivity (RD). AD, MD and RD are expressed in μm^2^.ms^−1^. *ML* molecular layer, *SLM* stratum lacunosum moleculare, *SR* stratum radiatum.

For each DTI dataset, Fig. 4 shows the 3D scatterplots of the 45 VOIs (15 for ML, 15 for SLM and 15 for SR) according to FA, MD and RD with a color-encoding either according to their true anatomical location, or to the resulting label of the unsupervised k-medoid clustering. Because the AD demonstrated less discrimination between the three hippocampal layers according to the previous post-hoc tests, we did not use this metrics for the representations.

Whatever the dataset, the points (i.e. VOIs) corresponding to the ML were better distinguished from those of the SLM and the SR, particularly with the B2700-43Dir dataset, which perfectly captured this layer. However, the points corresponding to the SLM and SR were more mixed whatever the dataset although there were less misclassifications with the B2700 datasets. Table 3 shows the ARIs for each DTI dataset. This translated into similar amount of potential misclassifications with B1000 datasets compared to B2700 datasets when comparing the clustering results and the real anatomical classification but higher ARI with B2700-22Dir and B2700-43Dir, namely: 15 misclassifications with B1000-12Dir (ARI = 0.356), versus 14 with B1000-22Dir (ARI = 0.366), versus 14 with B2700-12Dir (ARI = 0.351), versus 13 with B2700-22Dir (ARI = 0.430) versus 14 with B2700-43Dir (ARI = 0.480).

**Table 3:** Values of the adjusted Rand index evaluating the external quality of the clustering in the five diffusion tensor imaging (DTI) datasets.

Dataset	Adjusted Rand index
B1000-12Dir	0.356
B1000-22Dir	0.366
B2700-12Dir	0.351
B2700-22Dir	0.430
B2700-43Dir	0.480

### Detection of the microstructural alteration induced by EAE in the molecular layer

**Figure 5:**
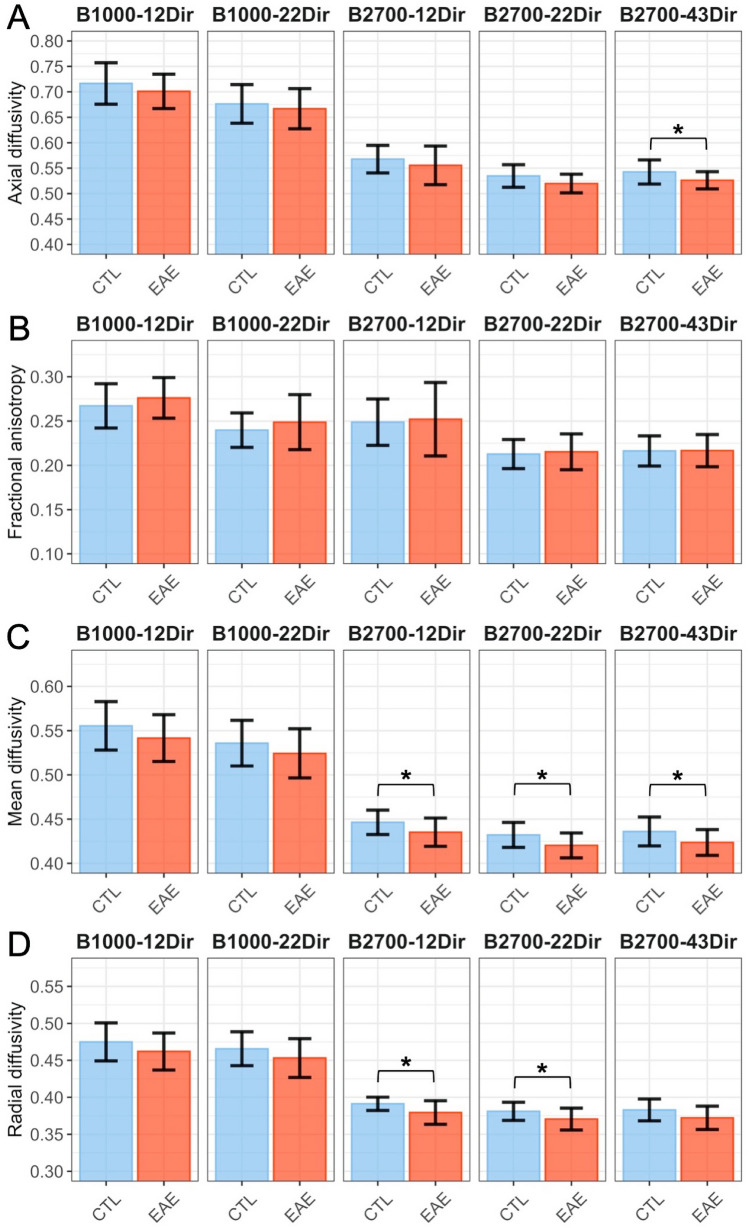
Comparisons between the diffusion tensor imaging (DTI) metrics from the five datasets in the molecular layer of control healthy mice (CTL) versus the mouse model of multiple sclerosis (experimental autoimmune encephalomyelitis [EAE]): (**A**) axial diffusivity, (**B**) fractional anisotropy, (**C**) mean diffusivity, (**D**) radial diffusivity. Diffusivities are expressed in μm^2^.ms^−1^. *P < 0.05. *B* b-value, *Dir* number of diffusion gradient directions.

Figure 5 and Supplementary Data S7 display the comparisons of the four DTI metrics for each DTI dataset and the DKI metrics between EAE and control mice in the molecular layer. There were trends towards lower AD, MD and RD values in EAE mice compared with control mice, but only the following comparisons reached significance: AD for the B2700-43Dir dataset (P = 0.0335), MD for the B2700-43Dir, B2700-22Dir and B2700-12Dir datasets (P = 0.0462, 0.0267 and 0.0332, respectively) and RD for the B2700-12Dir and B2700-22Dir datasets (P = 0.0180 and 0.0422, respectively). None of the comparisons involving the B1000 datasets were significant. Regarding the DKI computed using the full B1000 and B2700 shells, none of the DKI metrics was significantly different between EAE and control mice.

## Discussion

The ability to precisely and non-invasively capture the micro-structure of GM, either in cortex or subcortex, would be of great interest to better understand the brain’s normal functioning, maturation, and natural diversity, as well as the different stages of pathological processes, such as consequences of brain trauma, stroke to neurodegenerative and inflammatory diseases.

Paradoxically, the DTI, which was designed to model the anisotropic diffusion of water molecules along WM tract, provided very encouraging results in the GM^32–37^. Recent reports have highlighted the potential of DTI achieved at high b-values (> 2000 s.mm^−2^) to better characterize the GM microstructure^38,39^. However, the limits of fitting dMRI data with a Gaussian water diffusion model increases with high b-values^10^, which questions the adequacy of the DTI model in such conditions and necessitates validation of high b-values benefits for GM studies.

The investigation of GM using dMRI with strong diffusion-weightings is essentially limited by the relative decay of the magnetic-resonance signal with increasing b-value, and sometimes by the low spatial resolution of the achievable images. Thanks to the 3D-msEPI used in this study, a SNR of about 25—high enough for reliable estimation of diffusion metrics^14^, was measured in the hippocampus on mouse brain images acquired at high in-slice resolution (82 microns) and with strong diffusion-weighting (b = 2700 s.mm^−2^).

In agreement with the literature, this study demonstrated a consistent decrease in both the mean and directional diffusivities of the GM as the b-value increases^7^. That is probably due to the fact that dMRI with a high b-value is weighted by a sensitivity to water diffusion restrictions, which may reflect a more intracellular environment and is also less sensitive to the contribution of blood and cerebrospinal fluid (CSF) in DW-signal^40^.

While a few previous studies have reported a relative independence of FA from b-value intensity^7,8^, this work shows a consistent FA decrease in the GM as the b-value increases (Supplementary Data S6)^7,8^. Furthermore, all of the diffusion metrics of the GM were found to be sensitive to the number of diffusion directions encoded for each dataset. Such a dependence of DTI parameters on NDIR has been reported for WM ^41,42^.

In this study, we developed an original iterative process in order to select a desired number of diffusion gradient directions, based on the elimination of the vector with the largest number of vectors in the close neighborhood. This process was relatively easy to implement and provided coherent results during the visual and the quantitative quality controls. The minimum inter-vectors angles found after downsampling were not significantly different from those measured across the initial diffusion-encoding vectors. The average SNRs in the hippocampus were similar for datasets with the same b-value, and logically, decreased from the B0 image to the B1000 images (≈− 40%) and from the B1000 to the B2700 images (≈− 47%). Our initial MRI acquisition lasted 1 h and 50 min and artifacts, related to motion of the mice, led to the exclusion of 5/36 (14%) of the eligible population. Adding three other shells on the same MR-system would lead to protocol of 4h - 4h30 with high risk of poor quality data and additional exclusions. For this same reason, the number of diffusion directions was limited to 43 for b = 2700 s.mm^−2^ and to 22 for b = 1000 s.mm^−2^.

We also proposed an original method confronting five unsupervised classifications of the VOIs based on the DTI metrics coming from each DTI dataset, to their true anatomical label. We found advantage of using the highest available b-value and NDIR. The highest ARI was obtained with B2700-43Dir, which was the only dataset to perfectly capture the ML. Intermediate performances were obtained with the other B2700-22Dir datasets, while the lowest performances were obtained with the B1000 datasets and the B2700-12Dir. However, it should be noted that the SLM and SR layers were never perfectly distinguished, probably because of the similarity and low value of their isotropy.

Furthermore, we re-performed the comparisons between the DTI metrics of EAE versus control mice in the layer that is histologically affected at this stage of the EAE model, as in our seminal study^17^, but for each DTI dataset instead of only B2700-43Dir. Previously, decreases in MD and AD were correlated with the decrease in lengths and dendritic intersections, specifically in the ML of EAE mice as confirmed by histopathological analyses^17^. Importantly, the present study pointed out that the B2700-43Dir dataset was able to provide DTI metrics reflecting histopathological findings (i.e. significant decreases in MD and AD in the ML). Partial correlations were also found between the mouse groups and the diffusion metrics computed in the ML using the B2700-22Dir and B2700-12Dir datasets (significant decrease in MD with a clear downward trend of AD), as well as additional correlations (significant decrease in RD). In other words, the construction of reduced sets with fixed b-value led to a loss of sensibility of AD but not of MD for the detection of EAE effects. Hence, the MD in GM appeared more robust to the variation of NDIR. Complementary experiments (not shown) performed with a drastic reduction of the b = 2700 s.mm^−2^ sets to two sets of 6 diffusion encoding directions showed that the sensitivity to EAE was lost by the excessive removal of diffusion encoding directions. Finally, none of the B1000 datasets enabled to identify realistic hippocampal alterations.

In particular, the MD at b = 2700 s.mm^−2^ was the most sensitive DTI metrics to early decrease in dendritic length and their number of intersections in EAE mice in our study, which is in line with the strong correlation found between 1/MD and neurite density from the NODDI model in the human cortex at high b-values^37,38^. A first reason to explain the better performances, of B2700 datasets to capture hippocampal microstructure changes, could be the higher CNR found in the ML of B2700 datasets compared with B1000 datasets.

Although DTI are mostly acquired with low b-value (b ≤ 1000 s.mm^−2^), these parameters have not been shown to be specific to underlying microstructural features of neurites and are often related to water diffusion in tissue compartments other than neurites. Indeed, several studies have highlighted the increasing sensitivity of higher b-value DTI to neurites physiological and pathological changes because it could better capture the diffusion of slow-motion water molecule hindered between densely packed neurites and inside neurites and soma^43^.

Hui et al. demonstrated that the DTI sensitivity generally increases with high b-values for metrics measuring slow water molecule diffusivity (RD), and decreases for metrics measuring fast diffusivity (AD)^7^. In agreement with the findings by Hui et al., this study reveals that the sensitivity for monitoring GM microstructure changes of MD, AD, and RD, whose values in GM are significantly lower than that of AD measured in WM, is shown to be improved as b-value increases from 1000 to 2700 s.mm^−2^.

The increase in the DTI fit residue (SSE), which was driven in the hippocampus by increasing the b-value from 1000 to 2700 s.mm^−2^, is inferior to that observed in the WM of Corpus-Callosum (Supplemental Data S4). This could be an argument for using DTI with high b-values to explore gray matter. In the other hand, Kurtosis modeling was applied to dMRI data to assess the ability of a non-Gaussian component of the dMRI signal to detect the decrease in dendritic length and number of intersections in ML EAE mice. None of the DKI measures were significantly different between EAE and control hippocampus.

On a clinical and practical perspective, our results together with those of our initial study suggest that investigating subtle micro-structural changes in the cortex and the subcortex in neuroinflammatory diseases should attempt to maximize the b-value and to encode at least a moderate number (n = 22) of diffusion directions in one shell for DTI. Indeed, the acquisition protocol must last an acceptable duration for patients without omitting complementary sequences exploring inflammation, cytoarchitecture, myeloarchitecture and metabolism^17,38^. In addition, this statement can be refined according to the study by Fukutomi et al., which showed that DTI and NODDI provide redundant information in the GM but that DTI in GM should be acquired at high b-values because low b-values DTI is biased by heterogeneity and partial volume effect of cerebrospinal fluid. Conversely, DTI does not require exceedingly high NDIRs compared to NODDI (namely 30 directions are recommended versus 90 for NODDI)^38^.

Our study has limitations. First, the DTI datasets were reconstructed a posteriori from the initial B2700-43Dir and B1000-22Dir datasets. Thus, we did not prospectively investigate other NDIR and other b-values. Second, even if non-significant differences in the minimal angles were found between the initial and the sub-sampled 22 vectors and between the 12 vectors sub-sampled from the 43 and from the 22 initial vectors, the diffusion-encoding vectors used in this study were not strictly equivalent between B1000 and B2700 datasets. Indeed, the two shells were initially built to provide a uniform coverage on each shell as well as a global uniform coverage by using electrostatic repulsion^20,44^. Third, the thickness of the ROIs could be of one voxel only on some slice, which could have lead to partial volume effect and bias in the measurements—although we tried to minimize this bias by segmenting 3 contiguous slices for each ROI, and by double-checking all the ROIs. Fourth, the compromise established in this study between spatial and angular resolution, SNR, and acquisition time led to the coding of 43 diffusion directions. Although this number is lower than those achieved in some recent human dMRI studies^45^, it nevertheless remains sufficient for an accurate ROIs-based estimation of diffusion metrics^42^. Fifth, the spatial resolution was not isotropic (0.08 × 0.08 × 0.2 mm^3^) because we needed high-resolution coronal scanning (due to natural cylindrical shape of the mouse brain), and thicker slice covering the whole brain for successful co-registration and comparison with histology. However, this could have bias directionality-dependent metrics such as AD, RD and FA. Finally, The use of acceleration techniques, such as Simultaneous Multi-Slice acquisition, which is not currently available on scanners dedicated to small animals, would allow to reduce the total acquisition time of high b-value dMRI and/or increase the NDIR.

In summary, our study highlights that DTI performed with b = 2700 s.mm^−2^ appeared more sensitive than that collected with a standard b = 1000 s.mm^−2^ in distinguishing microarchitectures in each of the three layers of the hippocampus in healthy mice. Furthermore, only DTI with a high b-value was able to reveal the microstructural alterations, previously identified by histopathological analysis in the molecular layer of the hippocampus of EAE mice. At b = 2700 s.mm^−2^, the MD was found to be the most sensitive DTI metric to the early decreases in dendritic length and number of intersections occurring in the hippocampal molecular layer of EAE mice. Interestingly, axial and radial diffusivities were also able to reveal these microstructural alterations despite their greater sensitivity to NDIR. In contrast, FA and DKI metrics were totally unable to detect these microstructural changes. This study stresses the critical importance of the choice of b-value and NDIR to optimize the ability of DTI to capture brain microstructure as a function of the specific tissue type and targeted pathologies. It also highlights the caution needed in interpreting DTI indices.

## Supplementary Information

Supplementary Information.

## Data Availability

The datasets and the statistical analysis can be made available from the corresponding author on reasonable request.

## Abbreviations

AD: Axial diffusion
ANOVA: Analysis of variance
ARI: Adjusted rand index
ARRIVE: Animal Research: Reporting of In Vivo Experiments
CA: Cornus ammonis
CNR: Contrast to noise ratio
CSF: Cerebrospinal fluid
CTL: Control (sham) mice
D_ax_: Kurtosis-related axial diffusivity
DKE: Diffusion kurtosis estimator
DKI: Diffusion kurtosis imaging
D_rad_: Kurtosis-related radial diffusivity
D_mean_: Kurtosis-related mean diffusivity
dMRI: Diffusion MRI
DW: Diffusion weighted
DWI: Diffusion weighted imaging
DTI: Diffusion tensor imaging
EAE: Experimental autoimmune encephalomyelitis
EPI: Echo planner imaging
FA: Fraction of anisotropy
GM: Gray matter
K_ax_: Axial excess kurtosis
KFA: Kurtosis fractional anisotropy
K_mean_: Mean excess kurtosis
K_rad_: Radial excess kurtosis
MKT: Mean of the kurtosis tensor
ML: Molecular layer
MD: Mean diffusivity
MR: Magnetic resonance
Ms: Multi-shot
NDIR: Number of direction
NODDI: Neurite orientation dispersion and density imaging
SANDI: Soma and neurite density imaging
SD: Standard deviation
SI: Signal intensity
SLM: Stratum lacunosum moleculare
SNR: Signal to noise ratio
SR: Stratum radiatum
SSE: Sum of square error
RD: Radial diffusivity
VOI: Volume of interest
WM: White matter
