# Supplementary material for: High B-value diffusion tensor imaging for early detection of hippocampal microstructural alteration in a mouse model of multiple sclerosis
Source: Sci Rep. 2022 Jul 14;12:12008. doi: 10.1038/s41598-022-15511-0 (PMC9283448; doi:10.1038/s41598-022-15511-0)
Supplement: Supplementary file 1 — Supplementary Information. [file 41598_2022_15511_MOESM1_ESM.docx]

**SUPPLEMENTARY DATA**

**Supplementary Data S1.** Coordinates of the diffusion encoding vectors for each diffusion tensor imaging (DTI) dataset.

| **Dataset** | **x** | **y** | **z** |
| --- | --- | --- | --- |
| **B1000-12Dir** |  |  |  |
| 1 | 0.024989 | -0.664715 | 0.746679 |
| 2 | 0.363088 | 0.928226 | 0.08102 |
| 3 | 0.706186 | -0.706186 | 0.051013 |
| 4 | 0.699078 | 0.624069 | -0.349039 |
| 5 | 0.915571 | -0.283867 | -0.284867 |
| 6 | 0.721934 | -0.419961 | 0.549949 |
| 7 | 0.367005 | 0.414005 | -0.833011 |
| 8 | 0.130966 | -0.950751 | -0.280927 |
| 9 | -0.601231 | 0.44417 | 0.664255 |
| 10 | 0.942064 | -0.246017 | 0.228016 |
| 11 | 0.189022 | 0.552064 | 0.812094 |
| 12 | -0.537953 | 0.008999 | -0.842927 |
| **B1000-22Dir** |  |  |  |
| 1 | 0.97230146 | 0.190058927 | 0.13604218 |
| 2 | 0.024989269 | -0.664714566 | 0.74667937 |
| 3 | 0.221853846 | -0.724522697 | -0.652570098 |
| 4 | -0.681686156 | -0.065969628 | 0.728664527 |
| 5 | 0.363088241 | 0.928225586 | 0.08101969 |
| 6 | 0.706186105 | -0.706186105 | 0.051013444 |
| 7 | -0.053048349 | 0.18717059 | 0.980894002 |
| 8 | 0.699077602 | 0.624069276 | -0.349038745 |
| 9 | -0.232975073 | 0.944898901 | -0.229975394 |
| 10 | 0.558016462 | 0.569016786 | 0.604017819 |
| 11 | 0.915571155 | -0.283867039 | -0.284866571 |
| 12 | 0.721933585 | -0.419961365 | 0.549949407 |
| 13 | 0.367004771 | 0.414005382 | -0.833010829 |
| 14 | 0.130965757 | -0.950751411 | -0.280926547 |
| 15 | -0.601230616 | 0.444170372 | 0.664254791 |
| 16 | -0.776919981 | -0.605937592 | -0.17098239 |
| 17 | 0.201079845 | -0.281111623 | 0.938372608 |
| 18 | 0.942064063 | -0.24601673 | 0.228015506 |
| 19 | 0.267983921 | 0.869947805 | -0.413975162 |
| 20 | 0.189021833 | 0.552063767 | 0.812093802 |
| 21 | 0.911816743 | 0.202959209 | 0.356928265 |
| 22 | -0.5379532 | 0.008999217 | -0.842926669 |
| **B2700-12Dir** |  |  |  |
| 1 | 0.145918 | -0.040977 | -0.988448 |
| 2 | -0.908385 | -0.392166 | -0.14506 |
| 3 | -0.84229 | 0.425641 | 0.330721 |
| 4 | 0.23498 | 0.720939 | 0.651945 |
| 5 | -0.792944 | -0.116992 | 0.597958 |
| 6 | -0.725091 | 0.63808 | -0.259032 |
| 7 | 0.371882 | -0.706775 | -0.601808 |
| 8 | -0.266976 | -0.364968 | 0.891921 |
| 9 | 0.757946 | -0.262981 | -0.596958 |
| 10 | -0.363731 | -0.852369 | -0.37572 |
| 11 | -0.252983 | 0.522965 | 0.813945 |
| 12 | 0.491931 | -0.85388 | 0.169976 |
| **B2700-22Dir** |  |  |  |
| 1 | 0.145918 | -0.040977 | -0.988448 |
| 2 | 0.23498 | 0.720939 | 0.651945 |
| 3 | -0.792944 | -0.116992 | 0.597958 |
| 4 | 0.371882 | -0.706775 | -0.601808 |
| 5 | 0.046021 | -0.751345 | 0.658303 |
| 6 | -0.266976 | -0.364968 | 0.891921 |
| 7 | 0.508035 | -0.314021 | -0.802055 |
| 8 | 0.052011 | 0.956199 | 0.28806 |
| 9 | -0.573155 | 0.807218 | 0.141038 |
| 10 | 0.409951 | -0.74591 | 0.524937 |
| 11 | -0.019005 | -0.443127 | -0.896257 |
| 12 | -0.141078 | 0.424235 | -0.894495 |
| 13 | -0.538841 | -0.099234 | 0.836543 |
| 14 | 0.279198 | -0.895635 | -0.346246 |
| 15 | -0.331939 | -0.387929 | -0.859842 |
| 16 | 0.757946 | -0.262981 | -0.596958 |
| 17 | 0.118994 | -0.918955 | 0.375982 |
| 18 | -0.363731 | -0.852369 | -0.375722 |
| 19 | -0.780648 | 0.410341 | -0.471391 |
| 20 | -0.252983 | 0.522965 | 0.813945 |
| 21 | 0.221047 | 0.643135 | -0.733154 |
| 22 | 0.491931 | -0.85388 | 0.169976 |
| **B2700-43Dir** |  |  |  |
| 1 | 0.145918454 | -0.0409771 | -0.988447612 |
| 2 | -0.194670629 | 0.978396426 | -0.069597272 |
| 3 | -0.908384782 | -0.392166118 | -0.145061447 |
| 4 | -0.842290248 | 0.425641335 | 0.330721319 |
| 5 | -0.577795208 | 0.344877762 | -0.739737809 |
| 6 | 0.49718449 | 0.685254277 | -0.532197482 |
| 7 | 0.234980028 | 0.720938723 | 0.651944587 |
| 8 | -0.792943703 | -0.116991694 | 0.597957547 |
| 9 | -0.725090642 | 0.638079765 | -0.259032381 |
| 10 | -0.601036965 | -0.276016976 | -0.750046129 |
| 11 | 0.371881575 | -0.706774928 | -0.601808355 |
| 12 | 0.046021152 | -0.751345323 | 0.65830256 |
| 13 | -0.509758177 | -0.856593643 | -0.079962067 |
| 14 | 0.973183949 | -0.117022119 | 0.198037433 |
| 15 | -0.26697624 | -0.364967519 | 0.891920623 |
| 16 | 0.508034548 | -0.314021354 | -0.802054542 |
| 17 | 0.052010819 | 0.95619891 | 0.288059923 |
| 18 | -0.306079438 | 0.038009865 | -0.951246881 |
| 19 | 0.856528183 | 0.441756659 | -0.266853004 |
| 20 | -0.573155059 | 0.807218382 | 0.141038156 |
| 21 | -0.658029941 | -0.593026983 | -0.464021113 |
| 22 | 0.409950604 | -0.745910123 | 0.524936749 |
| 23 | -0.019005455 | -0.443127196 | -0.896257263 |
| 24 | 0.234076204 | 0.927301886 | -0.292095092 |
| 25 | 0.977946703 | -0.03999782 | -0.204988828 |
| 26 | -0.824325565 | 0.053955855 | -0.563538932 |
| 27 | -0.141078108 | 0.424234879 | -0.89449524 |
| 28 | -0.538840578 | -0.099233992 | 0.836542555 |
| 29 | 0.279198022 | -0.895635231 | -0.346245575 |
| 30 | 0.674033365 | 0.720035643 | -0.165008168 |
| 31 | -0.894876963 | 0.444938825 | -0.034995188 |
| 32 | -0.331938929 | -0.387928628 | -0.859841804 |
| 33 | 0.757946188 | -0.262981329 | -0.596957618 |
| 34 | 0.118994169 | -0.918954972 | 0.375981577 |
| 35 | -0.857925364 | -0.266976774 | -0.438961812 |
| 36 | 0.603021408 | 0.436015479 | -0.668023715 |
| 37 | 0.363730757 | -0.852369054 | -0.375721881 |
| 38 | -0.780647816 | 0.410340519 | -0.471391181 |
| 39 | -0.252983051 | 0.522964963 | 0.813945467 |
| 40 | 0.221046535 | 0.643135394 | -0.733154345 |
| 41 | 0.491931134 | -0.853880465 | 0.169976205 |
| 42 | 0.976015616 | 0.204003264 | -0.076001216 |
| 43 | -0.635107342 | 0.634107173 | 0.441074548 |

**Supplementary Data S2.** An example of parametric maps of Diffusion Kurtosis Imaging (DKI) ; DKI-related fractionnal anisotropy (FA), axial diffusivity (D_ax_), radial diffusivity (D_rad_), mean diffusivity (D_mean_), axial kurtosis (K_ax_), radial kurtosis (K_rad_), mean kurtosis (K_mean_), mean of the kurtosis tensor (MKT), Kurtosis Fractionnal Anisotropy (KFA), and sum of squared residual (SSR), also called kurtosis fit residue.


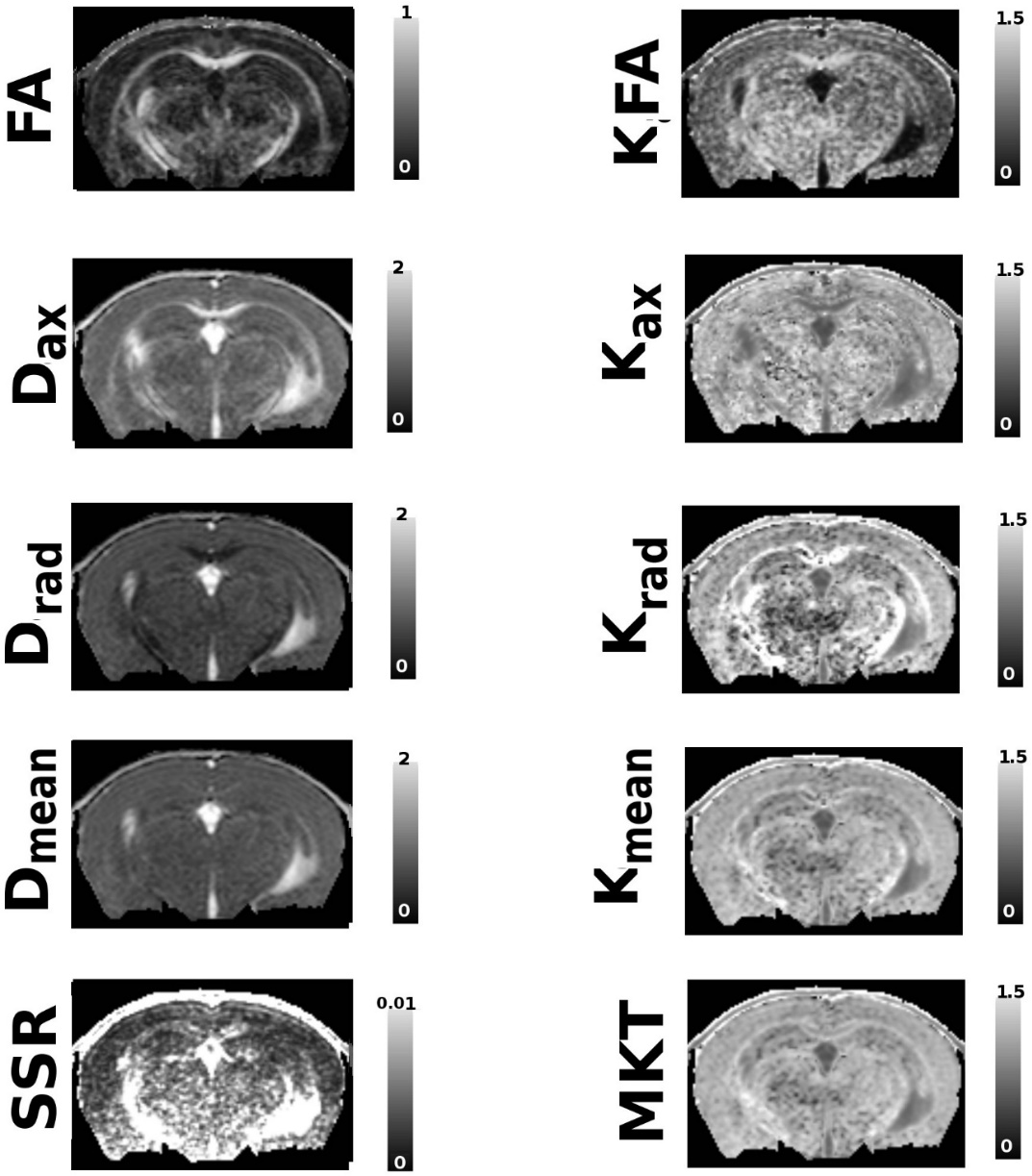


**Supplementary Data S3.** Visual representations of the posthoc comparisons regarding (**A**) the minimal angles between diffusion gradient vectors (unpaired t-test or Wilcoxon test, as appropriate, with adjustment for multiple comparisons), (**B**) the signal-to-noise ratio (SNR) in the hippocampus, and the contrast-to-noise ratio (CNR) in the (**C**) hippocampus, (**D**) stratum radiatum (SR), (**E**) stratum lacunosum molecular (SLM) and (**F**) molecular layer (ML). For SNRs and CNRs, tests are homo- or heteroscedastic paired t-test, or paired Wilcoxon test, as appropriate, with adjustment for multiple comparisons. *: P<0.05; **: P < 0.005; ***: P < 0.001.


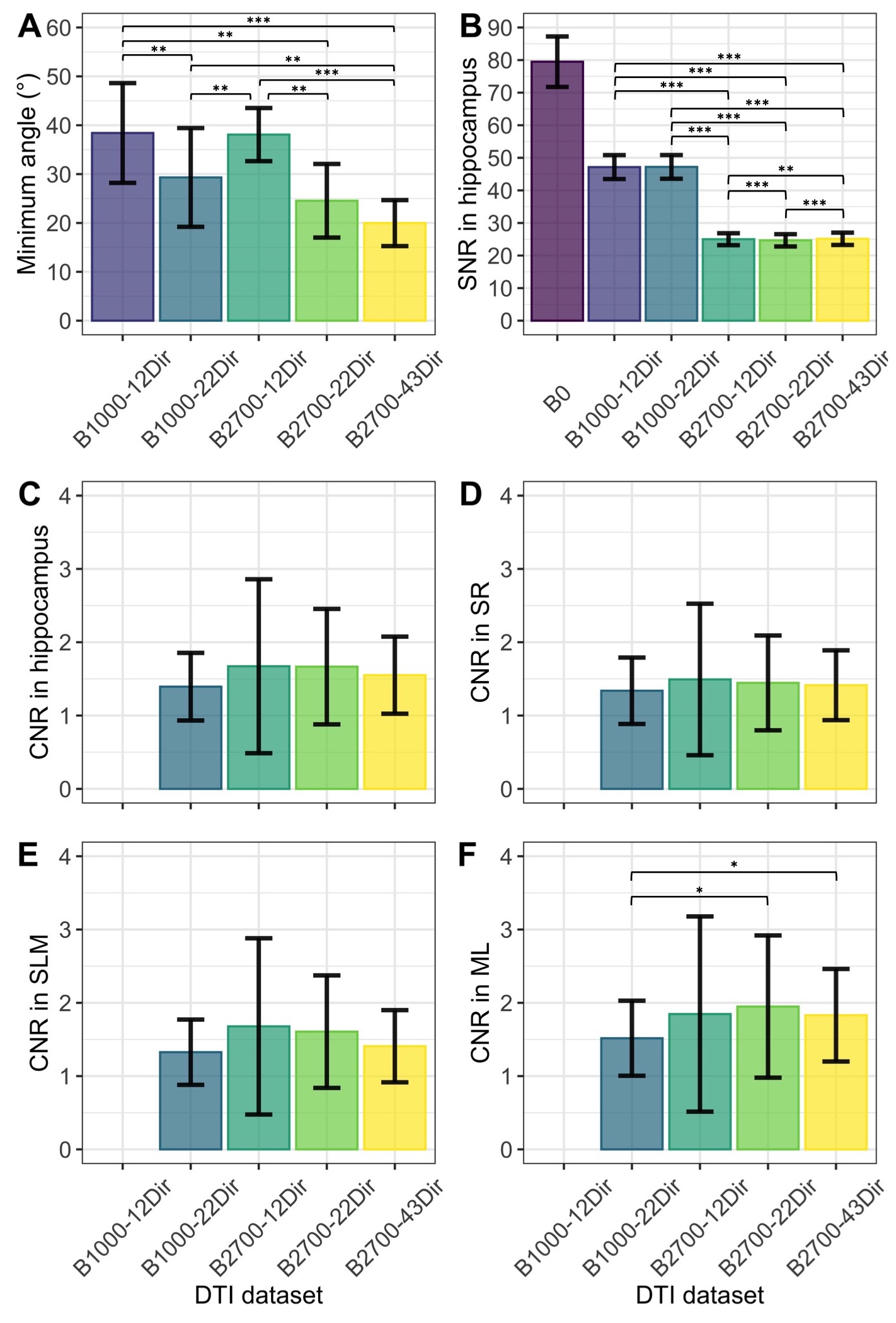


**Supplementary Data S4.** Sum of the square error (SSE) depending on the DTI datasets for the white matter (WM, i.e. corpus callosum) and the gray matter (GM, herein, the hippocampus). Unless specified in red (n.s.: not significant), all the post-hoc tests were significant.


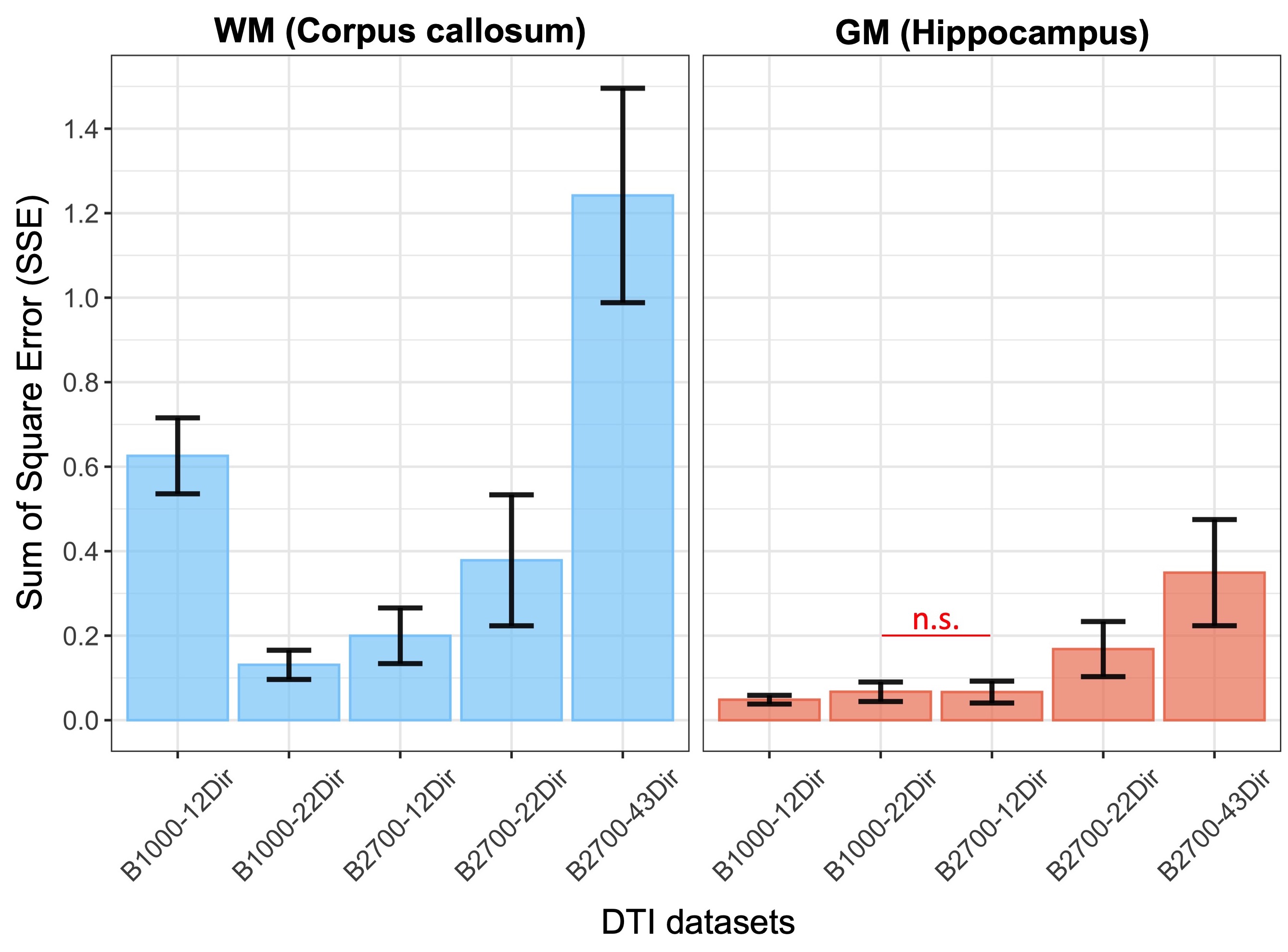


**Supplementary Data S5.** Assessment of the influence of the diffusion tensor imaging (DTI) dataset on the DTI metrics in each layer, in the control group (healthy mice).

| **DTI metrics** | **Layer** | **Datasets** | | | | | **F-value** | **Size effect** | **P-value** |
| --- | --- | --- | --- | --- | --- | --- | --- | --- | --- |
|  |  | **B1000-12Dir** | **B1000-22Dir** | **B2700-12Dir** | **B2700-22Dir** | **B2700-43Dir** |  |  |  |
| AD | ML | 0.716 ± 0.041 | 0.676 ± 0.038 | 0.568 ± 0.027 | 0.535 ± 0.022 | 0.542 ± 0.024 | 216.42 | 0.859 | <0.0001*** |
|  | SR | 0.699 ± 0.036 | 0.662 ± 0.036 | 0.563 ± 0.033 | 0.521 ± 0.024 | 0.53 ± 0.025 | 60^§^ | 1 | <0.0001*** |
|  | SLM | 0.715 ± 0.034 | 0.683 ± 0.028 | 0.546 ± 0.027 | 0.519 ± 0.022 | 0.519 ± 0.022 | 417.39 | 0.914 | <0.0001*** |
| RD | ML | 0.475 ± 0.026 | 0.466 ± 0.023 | 0.391 ± 0.009 | 0.381 ± 0.012 | 0.383 ± 0.015 | 51.57^§^ | 0.860 | <0.0001*** |
|  | SR | 0.528 ± 0.026 | 0.517 ± 0.023 | 0.438 ± 0.018 | 0.43 ± 0.022 | 0.432 ± 0.022 | 53.06^§^ | 0.884 | <0.0001*** |
|  | SLM | 0.535 ± 0.032 | 0.518 ± 0.029 | 0.426 ± 0.019 | 0.417 ± 0.022 | 0.423 ± 0.022 | 291.20 | 0.819 | <0.0001*** |
| MD | ML | 0.555 ± 0.027 | 0.536 ± 0.026 | 0.446 ± 0.014 | 0.432 ± 0.014 | 0.436 ± 0.016 | 191.34 | 0.880 | <0.0001*** |
|  | SR | 0.585 ± 0.028 | 0.565 ± 0.026 | 0.474 ± 0.022 | 0.46 ± 0.022 | 0.465 ± 0.023 | 59.25^§^ | 0.988 | <0.0001*** |
|  | SLM | 0.595 ± 0.031 | 0.573 ± 0.028 | 0.464 ± 0.023 | 0.451 ± 0.021 | 0.455 ± 0.022 | 59.25^§^ | 0.988 | <0.0001*** |
| FA | ML | 0.267 ± 0.025 | 0.24 ± 0.019 | 0.249 ± 0.026 | 0.213 ± 0.016 | 0.216 ± 0.017 | 24.79 | 0.497 | <0.0001*** |
|  | SR | 0.194 ± 0.019 | 0.173 ± 0.016 | 0.187 ± 0.024 | 0.133 ± 0.011 | 0.139 ± 0.011 | 53.25 | 0.694 | <0.0001*** |
|  | SLM | 0.203 ± 0.022 | 0.193 ± 0.021 | 0.181 ± 0.016 | 0.157 ± 0.019 | 0.144 ± 0.019 | 50.77^§^ | 0.846 | <0.0001*** |

NOTE.- Metrics are expressed as mean ± standard deviation. F-values and P-values correspond to one-way repeated-measures analysis of variance of non-parametric Friedman test (§).

Diffusivities are expressed in μm^2^.ms^-1^.

Abbreviations: B: b-value, AD: axial diffusivity, Dir: number of diffusion gradient directions, FA: fractional anisotropy, MD: mean diffusivity, ML: molecular layer, RD: radial diffusivity, SLM: stratum lacunosum moleculare, SR: stratum radiatum.

*: P < 0.05, **: P < 0.005; ***: P < 0.001.

**Supplementary Data S6.** Comparison of the fractional anisotropy depending on the DTI datasets, for each layer.

Abbreviations: ML: molecular layer, SR: stratum radiatum, SLM: stratum lacunosum moleculare.

Post-hoc tests P-values: n.s.: P≥0.05; *: P<0.05; **: P<0.005; ***: P<0.001.


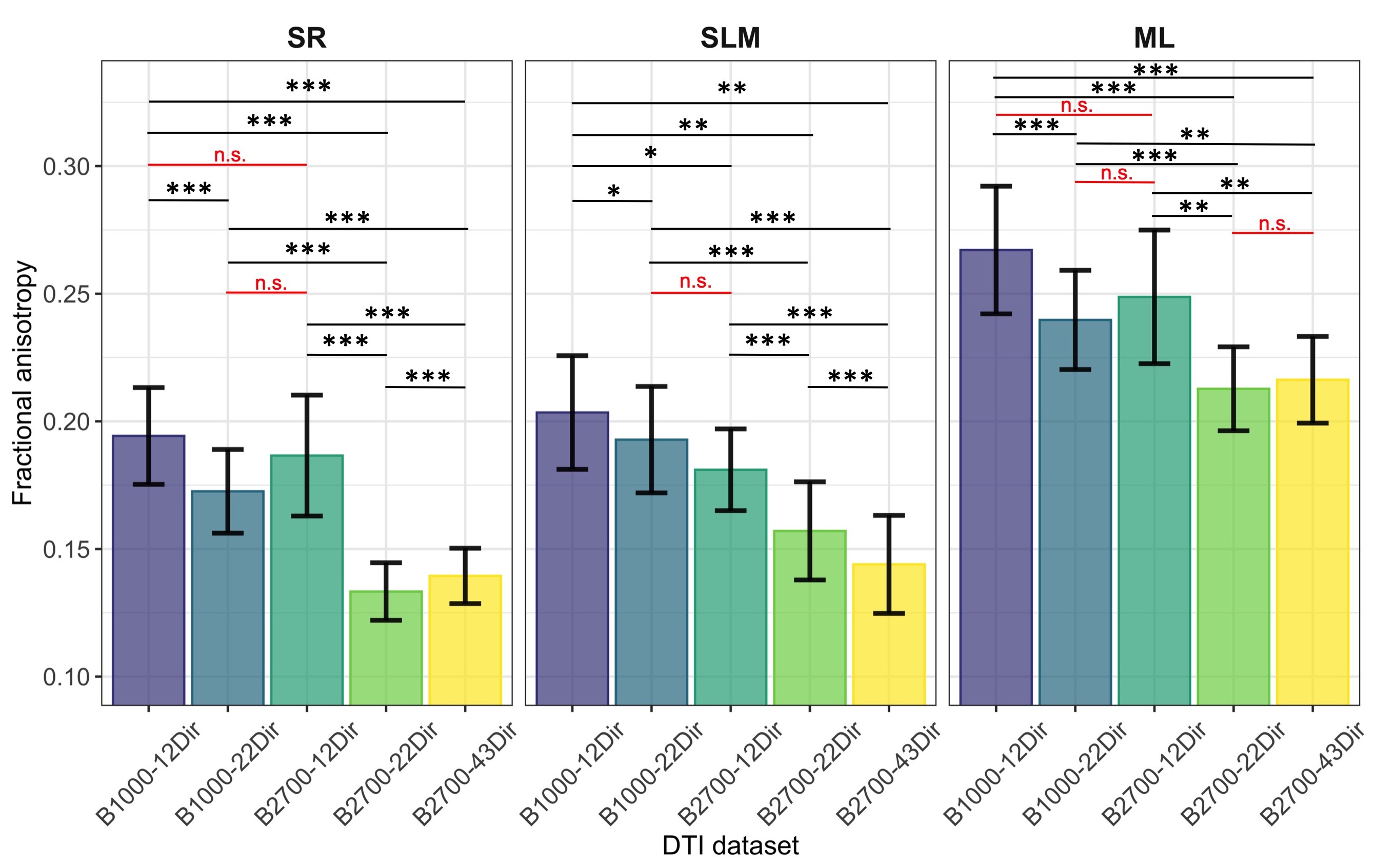


**Supplementary Data S7.** Comparisons of the diffusion tensor imaging (DTI) metrics and diffusion kurtosis imaging (DKI) metrics between control and experimental autoimmune encephalomyelitis (EAE) mice in the molecular layer (ML), for each dataset.

| **Data** | **Metrics** | **Mean +/- sd in the ML of CTL mice** | **Mean +/- sd in the ML of EAE mice** | **P-value** |
| --- | --- | --- | --- | --- |
| DKI | FA | 0.268 +/- 0.025 | 0.274 +/- 0.031 | 0.5553 |
|  | MD | 1.308 +/- 0.078 | 1.275 +/- 0.084 | 0.2646 |
|  | Dax | 0.788 +/- 0.052 | 0.77 +/- 0.055 | 0.3692 |
|  | Drad | 0.52 +/- 0.033 | 0.504 +/- 0.035 | 0.2075 |
|  | Kax | 0.924 +/- 0.087 | 0.954 +/- 0.131 | 0.4716 |
|  | Krad | 0.913 +/- 0.16 | 0.92 +/- 0.117 | 0.8793 |
|  | MKT | 0.912 +/- 0.125 | 0.927 +/- 0.095 | 0.708 |
|  | Kmean | 0.942 +/- 0.118 | 0.962 +/- 0.099 | 0.618 |
| DTI B1000-12Dir | FA | 0.267 +/- 0.025 | 0.276 +/- 0.023 | 0.3028 |
|  | MD | 0.555 +/- 0.027 | 0.542 +/- 0.026 | 0.1647 |
|  | AD | 0.716 +/- 0.041 | 0.701 +/- 0.034 | 0.258 |
|  | RD | 0.475 +/- 0.026 | 0.462 +/- 0.025 | 0.1671 |
| DTI B1000-22Dir | FA | 0.24 +/- 0.019 | 0.249 +/- 0.031 | 0.3438 |
|  | MD | 0.536 +/- 0.026 | 0.524 +/- 0.028 | 0.2441 |
|  | AD | 0.676 +/- 0.038 | 0.667 +/- 0.039 | 0.5012 |
|  | RD | 0.466 +/- 0.023 | 0.453 +/- 0.026 | 0.1674 |
| DTI B2700-12Dir | FA | 0.249 +/- 0.026 | 0.252 +/- 0.041 | 0.794 |
|  | MD | 0.446 +/- 0.014 | 0.435 +/- 0.016 | **0.0462*** |
|  | AD | 0.568 +/- 0.027 | 0.556 +/- 0.038 | 0.0655§ |
|  | RD | 0.391 +/- 0.009 | 0.379 +/- 0.016 | **0.018*** |
| DTI B2700-22Dir | FA | 0.213 +/- 0.016 | 0.215 +/- 0.02 | 0.7025 |
|  | MD | 0.432 +/- 0.014 | 0.42 +/- 0.014 | **0.0267*** |
|  | AD | 0.535 +/- 0.022 | 0.52 +/- 0.018 | 0.051 |
|  | RD | 0.381 +/- 0.012 | 0.371 +/- 0.015 | **0.0422*** |
| DTI B2700-43Dir | FA | 0.216 +/- 0.017 | 0.217 +/- 0.018 | 0.9543 |
|  | MD | 0.436 +/- 0.016 | 0.424 +/- 0.015 | **0.0332*** |
|  | AD | 0.542 +/- 0.024 | 0.526 +/- 0.017 | **0.0335*** |
|  | RD | 0.383 +/- 0.015 | 0.372 +/- 0.016 | 0.0615 |

NOTE.- Metrics are expressed as mean ± standard deviation (sd). Tests are unpaired homo- or heteroscedastic t-tests or unpaired Wilcoxon tests (§) as appropriate.

*: P < 0.05. Significant results are in bold. Diffusivities are expressed in μm^2^.ms^-1^.
